# High-throughput assay exploiting disorder-to-order conformational switches: application to the proteasomal Rpn10:E6AP complex†

**DOI:** 10.1039/d3sc06370d

**Published:** 2024-02-06

**Authors:** Christine S. Muli, Sergey G. Tarasov, Kylie J. Walters

**Affiliations:** a Protein Processing Section, Center for Structural Biology, Center for Cancer Research, National Cancer Institute, National Institutes of Health Frederick MD 21702 USA kylie.walters@nih.gov; b Biophysics Resource, Center for Structural Biology, Center for Cancer Research, National Cancer Institute, National Institutes of Health Frederick MD 21702 USA

## Abstract

Conformational switching is pervasively driven by protein interactions, particularly for intrinsically disordered binding partners. We developed a dually orthogonal fluorescence-based assay to monitor such events, exploiting environmentally sensitive fluorophores. This assay is applied to E3 ligase E6AP, as its AZUL domain induces a disorder-to-order switch in an intrinsically disordered region of the proteasome, the so-named Rpn10 AZUL-binding domain (RAZUL). By testing various fluorophores, we developed an assay appropriate for high-throughput screening of Rpn10:E6AP-disrupting ligands. We found distinct positions in RAZUL for fluorophore labeling with either acrylodan or Atto610, which had disparate spectral responses to E6AP binding. E6AP caused a hypsochromic shift with increased fluorescence of acrylodan-RAZUL while decreasing fluorescence intensity of Atto610-RAZUL. Combining RAZUL labeled with either acrylodan or Atto610 into a common sample achieved robust and orthogonal measurement of the E6AP-induced conformational switch. This approach is generally applicable to disorder-to-order (or *vice versa*) transitions mediated by molecular interactions.

## Abstract 2

An assay was developed to monitor a disorder-to-order switch in proteasome subunit Rpn10 that is induced by E6AP binding. Fluorophores acrylodan and Atto610 differentially measured E6AP-induced helicity in Rpn10 RAZUL, allowing for dual application.

## Graphical abstract

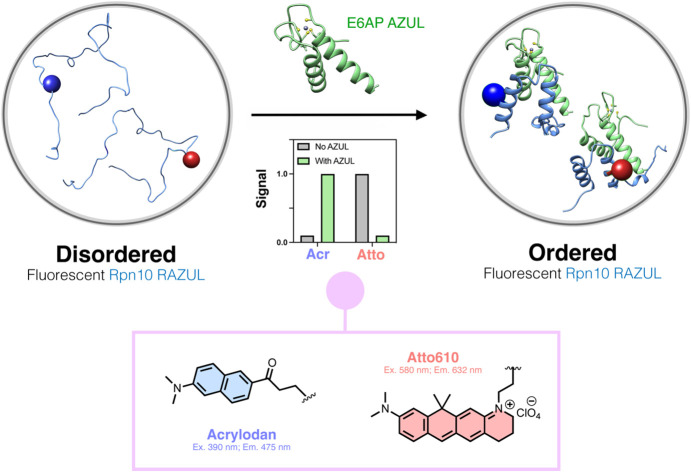

## Introduction

E3 ligases are critical for the transfer of ubiquitin to a protein substrate for ubiquitin-dependent proteasomal degradation.^1^ The 26S proteasome is a sophisticated multi-catalytic enzyme that recognizes ubiquitinated substrates with its 19S regulatory particle (RP), with subsequent degradation within its 20S core particle (CP).^2^ For targeted protein degradation, the activity of E3 ligases is therapeutically harnessed for development of proteolysis targeting chimeras (PROTACs), which act as heterobifunctional molecules that tether a substrate-of-interest to an E3, thereby inducing its ubiquitination.^3–7^ Akin to the PROTAC mechanism, E3 ligase E6AP/*UBE3A* is seized by the human papillomavirus (HPV) oncoprotein E6 to induce ubiquitination and subsequent degradation of tumor suppressor p53, driving HPV-associated cervical cancers.^8–12^ E6AP dysfunction is also implicated in a variety of other cancers, including prostate and breast cancer.^13–16^ Moreover, overexpression or gain-of-function of the *UBE3A* gene is linked to autism spectrum disorders,^17–20^ and loss or loss-of-function of *UBE3A* drives the neurological disorder Angelman syndrome.^21–24^ E6AP is present at the 26S proteasome^18,25^ by direct binding to the RP subunit Rpn10,^26^ which also regulates E6AP subcellular localization.^27–29^

To date, E6AP is the only E3 ligase with a known proteasomal binding site. The E6AP AZUL (amino-terminal zinc-binding domain of ubiquitin E3a ligase) domain binds to an intrinsically disordered region in Rpn10 to induce a disorder-to-order transition in the so-named Rpn10 AZUL-binding domain (RAZUL).^26^ With its prevalent role in cancer and neurological disease and with the importance of E3s for targeted protein degradation, E6AP is an attractive therapeutic target. Chemical probes against the Rpn10:E6AP interaction would be useful for interrogating the physiological significance of the E6AP interaction with proteasomes and the contribution of this interaction to E6AP-associated diseases. Several E6AP-interacting small molecules have been identified that bind to domains other than the AZUL domain or at unknown sites.^30–32^ A ligand that blocks Rpn10 binding to K48-linked ubiquitin chains was also reported,^33^ but this outcome is independent of the RAZUL domain.

**Fig. 1:**
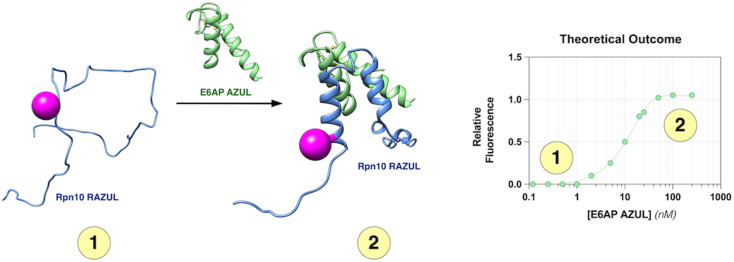
Assay design to monitor Rpn10 RAZUL binding to E6AP AZUL. RAZUL (blue) is intrinsically disordered (1) but becomes helical upon interaction with AZUL (green) to form an intermolecular 4-helix bundle (2). An environmentally sensitive fluorophore (magenta) is expected to have distinct signals for the (1) free and (2) bound states. PDB: 6U19.

Recently, environmentally sensitive fluorophores have proven useful as probes against systems that undergo conformational changes, including conformational switching during enzymatic activity^34^ and between intrinsically disordered tau and tubulin dimers.^35^ To develop an assay for the discovery of ligands that disrupt or enhance the Rpn10:E6AP interaction, we used environmentally sensitive fluorophores as sensors of the RAZUL disorder-to-order switch, Fig. 1. Such exploitation of a disorder-to-order transition of an intrinsically disordered protein (IDP) or region (IDR) is of therapeutic interest,^36–38^ especially when induced by a native binding partner; for example, p27 with Cdk2/cyclin A,^39,40^ MAX with cMyc,^41,42^ and p53 with MDM2.^43–45^ Thus, the approach we describe herein for Rpn10:E6AP, whereby binding is monitored by using environmentally sensitive fluorophores as sensors for conformational switching, can be applied generally to other therapeutically relevant protein systems to either characterize dynamic behavior and/or to perform high-throughput (HTP) screening for therapeutic discovery.

## Results and discussion

### Cysteine-substitutions in Rpn10 RAZUL do not disrupt E6AP binding or its disorder-to-order conformational switch

Rpn10 RAZUL spans amino acids 305–377 with no native cysteine. To allow for fluorogenic labeling, we rationally introduced a thiol by examining the Rpn10:E6AP structure^26^ for surface exposed serines. Three native serines (S337, S358, and S361) were identified in Rpn10 RAZUL proximal to but not within the E6AP AZUL interacting surface (Fig. S1A†). These serines are within regions that become helical by RAZUL binding to E6AP and are therefore likely to be sensitive to the RAZUL disorder-to-order transition. We tested and found that addition of the E6AP AZUL (24–87) domain induces helicity in RAZUL with cysteine substitution at S337, S358, or S361, with comparison to unmodified RAZUL. Specifically, experimentally measured circular dichroism (CD) spectra were compared to the theoretical sum of free RAZUL and AZUL to find induced helicity, with an enhanced effect for RAZUL S361C (Fig. S1B†). Based on these results, we concluded that AZUL induces helicity in all RAZUL mutants tested akin to wildtype RAZUL; however, we cannot preclude the possibility of the serine-to-cysteine mutations causing differences in the overall helical arrangements of mutant RAZUL when AZUL-bound. We therefore tested whether the binding affinity for AZUL is altered by these amino acid substitutions.

Previously, the binding affinity of the RAZUL:AZUL interaction was characterized by surface plasmon resonance (SPR) and isothermal titration calorimetry (ITC), and binding dissociation constants (*K*_d_) were agreeable at 11.6 ± 3.3 nM and 8.1 ± 1.4 nM, respectively.^26^ To test directly the impact of RAZUL cysteine substitution on AZUL binding affinity, *K*_d_ values were measured by ITC to find similarity with wildtype RAZUL and <30 nM affinity for all variants with AZUL (Fig. S1C†). The largest deviation was for RAZUL S361C at 6.90 ± 4.04 nM affinity. Altogether, these results indicate that cysteine substitution at codon 337, 358, or 361 does not affect Rpn10 RAZUL interaction with E6AP AZUL. Overall, the RAZUL cysteine mutants' retention of both the disorder-to-order transition by CD and binding interaction by ITC allowed for investigation of environmentally sensitive fluorophores with this system.

### Acrylodan labeling at Rpn10 RAZUL S358C is optimal for detecting E6AP binding

**Fig. 2:**
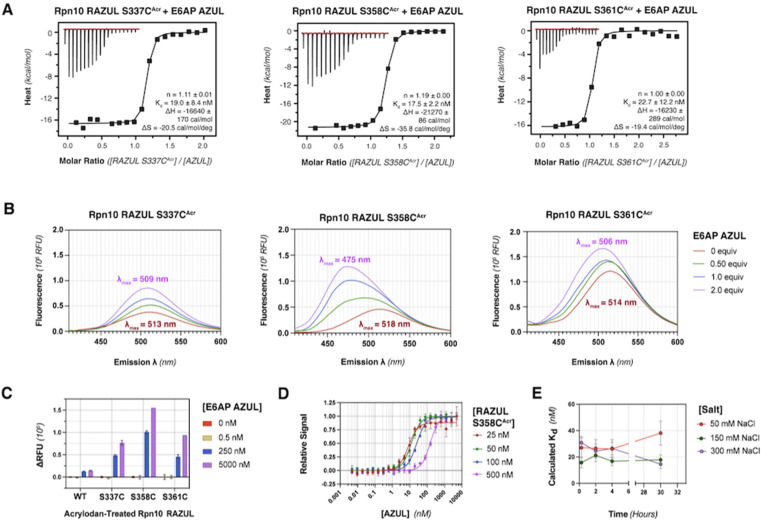
Acrylodan acts as an environmentally sensitive probe for Rpn10 RAZUL binding to E6AP AZUL, with greatest sensitivity for RAZUL S358C^Acr^. (A) Binding isotherm with raw ITC data (top left insert) for injections of E6AP AZUL into acrylodan-labeled Rpn10 RAZUL at 170 μM AZUL into 18 μM S337C^Acr^ (left panel), 90 μM AZUL into 9 μM S358C^Acr^ (middle panel), or 106 μM AZUL into 9 μM S361C^Acr^ (right panel). Fitted thermodynamic values are included on each panel at the bottom right: *n*, stoichiometry; *K*_d_, binding affinity; Δ*H*, change in enthalpy; Δ*S*, change in entropy. Measurements were made in 10 mM MOPS, pH 6.5, 50 mM NaCl, 2 mM TCEP, and 10 μM ZnSO_4_. (B) Fluorescence spectroscopy of 500 nM of RAZUL^Acr^ as indicated with 0–2.0 equivalences of E6AP AZUL in 10 mM MOPS, pH 6.5, 50 mM NaCl, 5 mM DTT, and 10 μM ZnSO_4_. All samples were excited at 390 nm on a FluoroMax-4C spectrofluorometer with emission scanning range from 410–600 nm. *λ*_max_ refers to the wavelength (*λ*) at which the maximum absorption is observed. (C) Change in RFU (relative to RFU_background_) for 500 nM of acrylodan-treated WT or cysteine-substituted Rpn10 RAZUL mixed with 0 (red), 0.5 (yellow), 250 (blue), or 5000 (purple) nM of E6AP AZUL (*n* = 3). A 96-well plate was used with excitation of 390 nm and emission of 500 nm. ΔRFU = RFU_signal_ − RFU_background_. (D) Fluorescence measurements of 25 (red), 50 (green), 100 (blue), or 500 (purple) nM RAZUL S358C^Acr^ with increasing quantities of E6AP AZUL normalized to RFU^max^ in a 384-well plate and buffer as in (B) but supplemented with 0.1% Tween 20 (*n* = 3). The relative signal was fit for specific binding to a Hill slope. (E) Fluorescence measurements of 100 nM RAZUL S358C^Acr^ mixed with 0–1500 nM E6AP AZUL following storage at 4 °C for 0.25, 2, 4, or 30 hours. Buffer conditions of (D) were used but with 50 (red), 150 (green), or 300 (purple) mM NaCl (*n* = 3). Curves were fit as in (D), and the respective *K*_d_ values were plotted against time. For (C) through (E), each data point is represented as mean ± standard error of the mean (SEM).

To produce fluorescently labeled RAZUL constructs, we treated cysteine-substituted and wildtype (negative control) RAZUL with 2-fold equivalent acrylodan to achieve >99% labeling efficiency of the mutants, as indicated by intact protein mass spectrometry (Fig. S2†). Acrylodan labeling did not interfere with the RAZUL:AZUL interaction, as each labeled RAZUL variant bound AZUL with <35 nM affinity by ITC (Fig. 2A). Each acrylodan-labeled Rpn10 RAZUL (RAZUL^Acr^) sample was analyzed by fluorescence spectroscopy to evaluate sensitivity to AZUL binding. In each case, the fluorescence of 500 nM Rpn10 RAZUL^Acr^ was measured with increasing quantities of E6AP AZUL up to 2 equivalences, recording with an excitation at 390 nm and emission scanning range of 410–600 nm. Relative fluorescent units (RFU) were plotted to find a dose-dependent hypsochromic shift for each RAZUL^Acr^ probe, with the maximum wavelength (*λ*_max_) of RAZUL S358C^Acr^ shifted from 518 nm to 475 nm (Fig. 2B). More minor shifts of 4 nm and 8 nm were measured for RAZUL S337C^Acr^ and S361C^Acr^, respectively (Fig. 2B). The RFU similarly increased dose-dependently with E6AP AZUL for each RAZUL^Acr^ mutant when the assay was translated to a 96-well format at 50 μL per well. The change in RFU (ΔRFU) was plotted with acrylodan-treated wildtype Rpn10 RAZUL (which was not labeled) used as a negative control (Fig. 2C). The affinity of RAZUL S358C^Acr^ for AZUL was measured to be similar to RAZUL S337C^Acr^ and S361C^Acr^ by ITC (Fig. 2A), indicating that the more prominent spectral shift in RAZUL S358C^Acr^ is driven by a greater change in the local chemical environment rather than by increased affinity. Since RAZUL S358C^Acr^ elicited the largest response, it was chosen for further optimization.

We next translated the acrylodan-based assay to a 384-well plate with 10–500 nM RAZUL S358C^Acr^ and sequentially increased quantities of AZUL. The relative fluorescent signal was normalized to the maximum RFU value, namely at saturating AZUL concentrations (Fig. 2D). The assay stability and optimal buffer conditions were assessed to find that without detergent, a decrease in signal occurred after the saturation point from protein aggregation (Fig. S3A†). All binding curves could be fit with an *R*^2^ > 0.96 when Tween 20 was included, whereas without it, the *R*^2^ values ranged from 0.83 to 0.94. To ensure the fluorescence profile of RAZUL S358C^Acr^ was not compromised by detergent, we evaluated free and bound RAZUL S358C^Acr^ with 0–0.1% Tween 20 and found no significant dependence (Fig. S3B and C†). We next measured the reproducibility of the assay at 4 °C with 50, 150 or 300 mM NaCl to find stability over 30 hours based on *K*_d_ measurements, with best results at 150 mM NaCl for a consistent *K*_d_ of ∼18 nM (Fig. 2E). Therefore, we concluded that RAZUL S358C^Acr^ conformational switching in response to AZUL binding could be measured in a 384-well plate format, with most stable signal when 150 mM NaCl and 0.1% Tween 20 are included in the buffer.

### RAZUL S358C^Acr^ detects competitive inhibition for E6AP AZUL in a HTP screening format

**Fig. 3:**
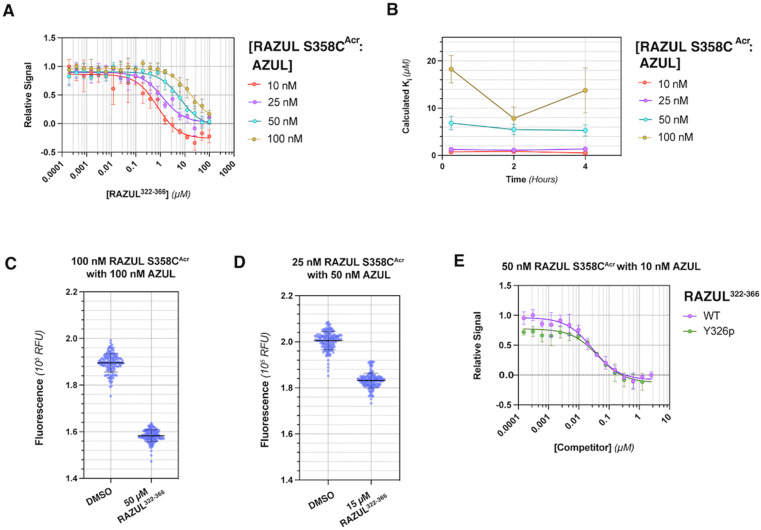
RAZUL S358C^Acr^ is sensitive to competition for AZUL by RAZUL^322–366^. (A) Plot of RFU values normalized by RFU^max^ for 10 (red), 25 (purple), 50 (cyan), or 100 (gold) nM RAZUL S358C^Acr^:AZUL against concentration of RAZUL^322–366^ (*n* = 3). Measurements were in 10 mM MOPS, pH 6.5, 150 mM NaCl, 5 mM DTT, 10 μM ZnSO_4_, 0.1% Tween 20 supplemented with 5% DMSO. Curves were fit to dose response with variable slope on GraphPad Prism. (B) Plot of *K*_i_ values for RAZUL^322–366^ after 0.25, 2, or 4 hours storage at 4 °C, based on fluorescent measurements of 10 (red), 25 (purple), 50 (cyan), or 100 (gold) nM RAZUL S358C^Acr^:AZUL, with the 0.25 hour timepoint as displayed in (A). Competition curves from different timepoints were plotted and fitted for *K*_i_ values, which are plotted against storage time. (C) RFU of 100 nM RAZUL S358C^Acr^:AZUL with DMSO (vehicle control) or 50 μM RAZUL^322–366^ in buffer supplemented with 1% BSA (*n* = 154). (D) RFU of 25 nM RAZUL S358C^Acr^:50 nM AZUL with DMSO (vehicle control) or 15 μM RAZUL^322–366^ in buffer supplemented with 1% BSA (*n* = 154). (E) Plot of RFU values normalized by RFU^max^ for 10 nM AZUL mixed with 50 nM RAZUL S358C^Acr^ against concentration of unmodified (purple) or Y326p (green) RAZUL^322–366^. Curves were fit to dose response with variable slope on GraphPad Prism. For all experiments, excitation and emission wavelengths of 390 nm and 475 nm were used, respectively. For (A), (B) and (E), each data point is represented as mean ± SEM.

To interrogate the application of RAZUL^Acr^ for identifying molecules that inhibit the Rpn10:E6AP interaction, we aimed to compete the most sensitive RAZUL^Acr^ probe (S358C^Acr^) off AZUL to simulate competitive inhibition. With no available chemical tools for the Rpn10:E6AP interaction, a synthetic RAZUL^322–366^ peptide with 18.2 ± 4.4 nM affinity for AZUL^26^ was used. Equimolar concentrations at 10, 25, 50, and 100 nM of RAZUL S358C^Acr^:AZUL were incubated with 0–100 μM RAZUL^322–366^ and ΔRFU values calculated, plotted, and fit to obtain an inhibition constant (*K*_i_). As expected, RAZUL^322–366^ competed with RAZUL S358C^Acr^ for AZUL, reducing the relative RAZUL S358C^Acr^ signal; greater quantities of RAZUL^322–366^ were required for competition when increasing concentration of RAZUL S358C^Acr^:AZUL (Fig. 3A). *K*_i_ values of 1.26 ± 0.28 μM and 18.2 ± 2.9 μM were measured for RAZUL^322–366^ at 25 nM and 100 nM RAZUL S358C^Acr^:AZUL, respectively. The slow off-rate of the RAZUL:AZUL interaction, which was previously found to be 2.11 ± 0.30 (×10^−2^ s^−1^),^26^ may cause the >800-fold increase in *K*_i_ compared to *K*_d_ at 25 nM RAZUL S358C^Acr^:AZUL. At 10–50 nM RAZUL S358C^Acr^:AZUL, the competition assay was stable over 4 hours at 4 °C, although less stability was detected at 100 nM RAZUL S358C^Acr^:AZUL (Fig. 3B).

To evaluate the applicability of this acrylodan-based assay for large scale HTP screening of Rpn10:E6AP-disrupting ligands, the fluorescence of 100 nM RAZUL S358C^Acr^:AZUL with DMSO (vehicle control) or 50 μM RAZUL^322–366^ (positive control for inhibition) was measured (Fig. 3C). Within this experiment, 1% carrier protein bovine serum albumin (BSA) was included in the buffer to increase stability.^46^ Notably, no overlap be Tween the positive and vehicle control measurements was observed. A basic Z′ factor of 0.37 was calculated from the average mean and standard deviation^47^ of the controls. For nanomolar interactions, as is the case for RAZUL:AZUL, quality of HTP assays have relied on the robust Z′ factor 
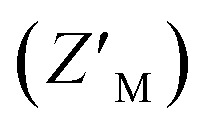
, which is calculated from the median and median absolute deviation.^48,49^ We calculated the 
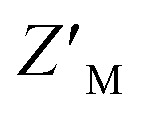
 to be 0.63, indicating robustness of the RAZUL S358C^Acr^ probe for competitive inhibition with AZUL binding. Decreasing amounts of material in the assay to 25 nM RAZUL S358C^Acr^:50 nM AZUL with or without 15 μM RAZUL^322–366^ reduces the 
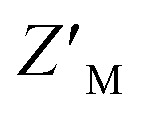
 to 0.35 (Fig. 3D).

We tested how this competitive assay performs with unsaturating conditions of AZUL with RAZUL S358C^Acr^ and whether it is sensitive to the 10-fold weaker affinity of phosphorylated RAZUL^322–366^ (Y326p) at 189.4 ± 43.8 nM.^26^ With 50 nM RAZUL S358C^Acr^ and 10 nM AZUL, *K*_i_ values of 28.5 ± 4.6 nM and 42.6 ± 13.1 nM were measured for unmodified and Y326p RAZUL^322–366^, respectively (Fig. 3E). These reduced values signify a greater sensitivity with sub-stoichiometric AZUL but their similarity indicates limited sensitivity to affinity differences in the sub-micromolar range. Moreover, these conditions yielded a poor 
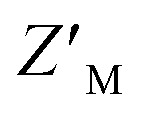
 factor, similar to Fig. 3D. Altogether, 100 nM RAZUL S358C^Acr^:AZUL was found to be acceptable for HTP applications (Fig. 3C) and even lower quantities of material are suitable for non-HTP evaluation of competitors (Fig. 3A and B). These results indicate that acrylodan-labeled RAZUL can serve as an environmental probe for the E6AP-induced disorder-to-order transition.

### Detection of E6AP binding by Atto610 is optimal at Rpn10 RAZUL S337C

**Fig. 4:**
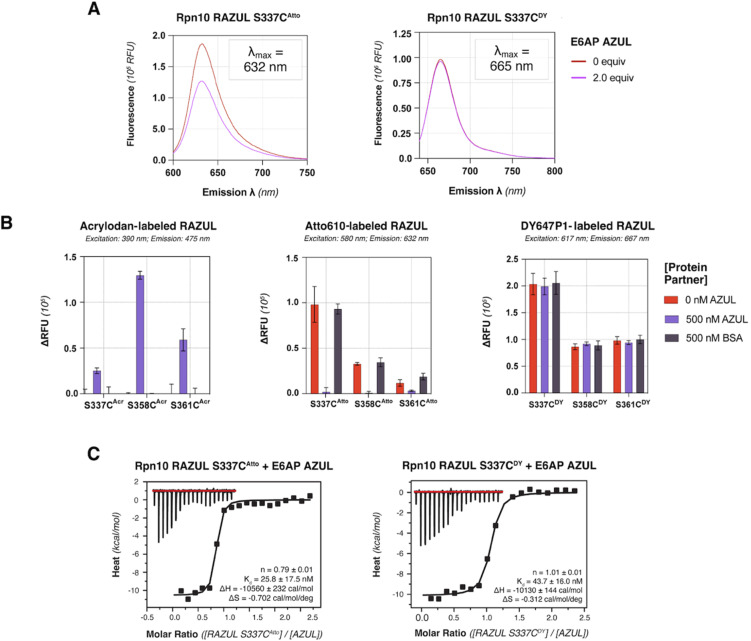
Evaluation of red-shifted fluorophores as probes for Rpn10 RAZUL binding to E6AP AZUL indicates greatest sensitivity for RAZUL S337C^Atto^. (A) Plot of RFU for 500 nM RAZUL S337C^Atto^ (left) or RAZUL S337C^DY^ (right) with 0 (red) or 2 (purple) equivalents of AZUL following excitation at 590 or 630 nm, respectively, with emission scanned from 600–750 nm or 640–800 nm, respectively, in 10 mM MOPS, pH 6.5, 50 mM NaCl, 5 mM DTT, and 10 μM ZnSO_4_. *λ*_max_ values are indicated. (B) Change in RFU (relative to RFU_background_) for 500 nM of cysteine-substituted RAZUL labeled with acrylodan (left), Atto610 (middle), or DY647P1 (right) mixed with 0 (red) or 500 (purple) nM AZUL or 500 nM BSA (dark grey) (*n* = 3). Measurements were recorded in a 384-well plate in 10 mM MOPS, pH 6.5, 150 mM NaCl, 5 mM DTT, 10 μM ZnSO_4_, and 0.1% Tween 20. (C) Binding isotherms with raw ITC data (top insert) for injections of 90 μM E6AP AZUL into 9 μM RAZUL S337C^Atto^ (left) or RAZUL S337C^DY^ (right) in 10 mM MOPS, pH 6.5, 50 mM NaCl, 2 mM TCEP, and 10 μM ZnSO_4_. Fitted thermodynamic values are included in the bottom right of each panel: *n*, stoichiometry; *K*_d_, binding affinity; Δ*H*, change in enthalpy; Δ*S*, change in entropy.

Based on its blue-shifted fluorescence, a major limitation for acrylodan as an environmental probe would be fluorescence overlap with potential compounds of interest.^50^ We therefore tested the red-shifted fluorophores Atto610 and DY647P1 by conjugating their maleimide-derivatives to the introduced cysteines of Rpn10 RAZUL. Conjugation conditions were optimized to >99% efficiency for 10 μM of cysteine-containing RAZUL mixed with 200 μM dye in 50 mM HEPES, 150 mM NaCl, pH 8, for 16–20 hours at 4 °C (Fig. S4 and S5†); insufficient and non-specific maleimide reactivity was measured at pH 7.4 and pH 8.5 respectively (Fig. S6†). The spectral profile of RAZUL S337C^Atto^ or RAZUL S337C^DY^ was measured without or with E6AP AZUL to find that the induced conformational switch causes an overall decrease in the emission scanning profile of RAZUL S337C^Atto^ (excitation at 590 nm) (Fig. 4A, left panel) with only a minor reduction for RAZUL S337C^DY^ (excitation at 630 nm) (Fig. 4A, right panel). In both cases, no change in *λ*_max_ was detected.

We next evaluated how dye-labeled RAZUL S337C, S358C, or S361C compared in plate reader assays following addition of E6AP AZUL or BSA (negative control). Consistent with (Fig. 2C), AZUL caused an increase in the fluorescent intensity of RAZUL^Acr^, with the largest increase for RAZUL S358C^Acr^ (excitation: 390 nm; emission: 475 nm), Fig. 4B (left panel). As expected, no change was observed with BSA. AZUL caused no change for RAZUL^DY^ (excitation: 617 nm; emission: 667 nm), Fig. 4B (right panel), indicating that the minor decrease by fluorescence emission scanning was not detectable in 384-well plate reader assays. RAZUL^Atto^ fluorescence, however, was reduced by AZUL (excitation: 580 nm; emission: 632 nm), with greatest effect for S337C^Atto^, (Fig. 4B, middle panel). The lack of effect for RAZUL^DY^ was not caused by loss of affinity for AZUL, as the binding constant of RAZUL S337C^DY^ and S337C^Atto^ for AZUL was similar by ITC measurement (Fig. 4C and S7†). Notably, all cysteine-substituted positions on RAZUL^Acr^ and RAZUL^Atto^ were sensitive to AZUL binding and could be used as fluorescent probes to monitor the disorder-to-order conformational switch. For further HTP assay optimization, RAZUL S358C was chosen as the most sensitive position for E6AP binding with acrylodan-labeling, whereas Atto610-labeling was most sensitive at RAZUL S337C.

**Fig. 5:**
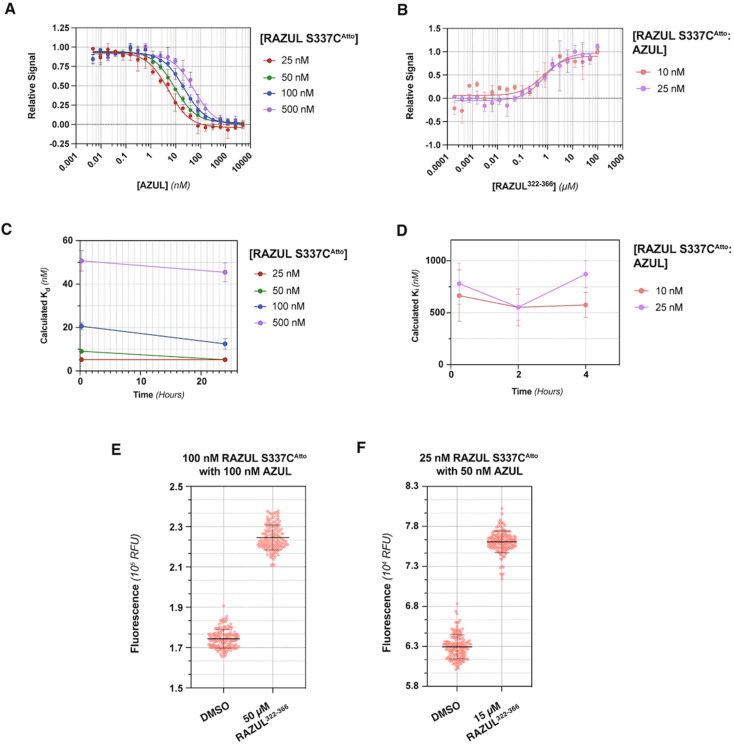
RAZUL S337C^Atto^ shows similar sensitivity as RAZUL S358C^Acr^ but with a reverse response. (A) Plot of RFU values normalized by RFU^max^ for 25 (red), 50 (green), 100 (blue), or 500 (purple) nM RAZUL S337C^Atto^ with varying quantities of AZUL in a 384-well plate and 10 mM MOPS, pH 6.5, 150 mM NaCl, 5 mM DTT, 10 μM ZnSO_4_, and 0.1% Tween 20 (*n* = 3). (B) Plot of RFU values normalized by RFU^max^ for 10 (red) or 25 (purple) nM S358C^Atto^:AZUL against varying concentrations of RAZUL^322–366^ in the buffer of (A) supplemented with 5% DMSO (*n* = 3). (C) Plot of *K*_d_ values after 0.25 or 24 hours storage at 4 °C, with the 0.25 hour timepoint based on fluorescent measurements from (A). 25 (red), 50 (green), 100 (blue), or 500 (purple) nM RAZUL S337C^Atto^ was prepared with 0–5000 nM E6AP AZUL in 10 mM MOPS, pH 6.5, 150 mM NaCl, 5 mM DTT, 10 μM ZnSO_4_, 0.1% Tween 20 (*n* = 3). After initial read of fluorescent values (shown in (A)), samples were stored at 4 °C and read by the CLARIOstar plate reader after 24 hours. (D) Plot of RAZUL^322–366^*K*_i_ values after 0.25, 2, or 4 hours storage at 4 °C based on fluorescence measurements of 10 (red) or 25 (purple) nM RAZUL S337C^Atto^:AZUL, with the 0.25 hour timepoint displayed in (B). The competition curves were plotted and fitted for *K*_i_ values. Those *K*_i_ values are plotted against storage time. In (A) through (D) the curves were fit in GraphPad Prism by dose response with variable slope and each data point is represented as mean ± SEM. (E) RFU for DMSO *versus* 50 μM RAZUL^322–366^ with 100 nM S337C^Atto^:AZUL (*n* = 154) in the buffer of (A) supplemented with 1% BSA and 5% DMSO. (F) RFU of 25 nM S337C^Atto^:50 nM AZUL with DMSO (vehicle control) or 15 μM RAZUL^322–366^ in buffer supplemented with 1% BSA and 5% DMSO (*n* = 154). For all experiments, excitation and emission wavelengths of 580 nm and 632 nm were used, respectively.

Evaluation of RFU with increasing quantities of AZUL for RAZUL S337C^Atto^ (Fig. 5A) in comparison to RAZUL S358C^Acr^ (Fig. 2D), with normalization to RFU^max^, indicated similar sensitivity, but with a reversal in signal. Inflection points in plots of the normalized RFU signals for increasing concentration of AZUL ranged from 5–55 nM in assays with 25–500 nM RAZUL S337C^Atto^ (Fig. 5A), resembling its binding affinity with E6AP AZUL (Fig. 4C, left panel). Addition of unlabeled RAZUL^322–366^ to 10 or 25 nM RAZUL S337C^Atto^:AZUL recovered the RFU signal (Fig. 5B) with a *K*_i_ of 0.664 ± 0.248 or 0.780 ± 0.199 μM, respectively. This *K*_i_ is less than the 1.26 ± 0.28 μM value for 25 nM RAZUL S358C^Acr^ with AZUL, indicating that less RAZUL^322–366^ is required to compete with 25 nM RAZUL S337C^Atto^ for AZUL compared to RAZUL S358C^Acr^. Moreover, binding (Fig. 5C) and competitive (Fig. 5D) assays were stable over time. We measured a 
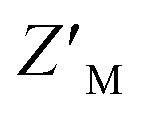
 factor for DMSO and 50 μM RAZUL^322–366^ of 0.57 with 100 nM RAZUL S337C^Atto^:AZUL (Fig. 5E). Further reduction of material using 25 nM RAZUL S337C^Atto^:50 nM AZUL with and without 15 μM RAZUL^322–366^ maintained a similar 
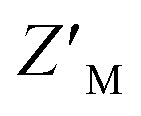
 of 0.60 (Fig. 5F). Unlike RAZUL S358C^Acr^ (Fig. 3D), the RAZUL S337C^Atto^ response by the E6AP-induced conformational switch is sensitive enough to obtain robust 
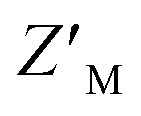
 factors at lower quantities, which would be advantageous for HTP screening.

**Fig. 6:**
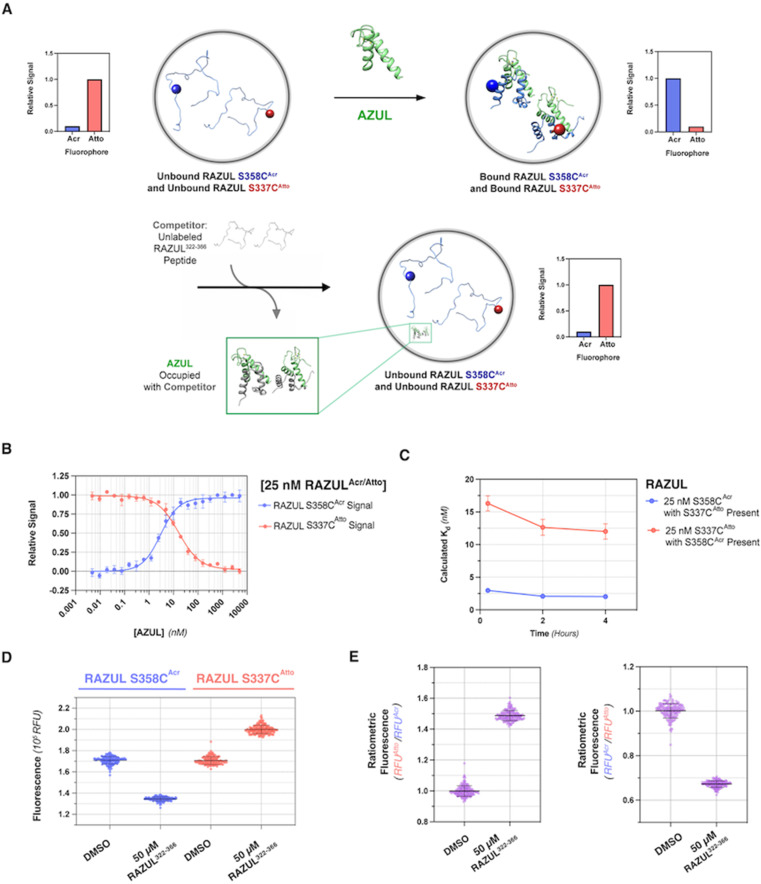
Dual application of RAZUL S358C^Acr^ and RAZUL S337C^Atto^ yields an orthogonal assay with sensitivity appropriate for HTP screening. (A) Concept of an orthogonal fluorescent-based assay with RAZUL S358C^Acr^ and RAZUL S337C^Atto^ in a common well with E6AP AZUL yielding opposite RFU response to the RAZUL disorder-to-order conformational switch. With the introduction of a competitor (such as positive control RAZUL^322–366^), the fluorescent probes are expected to have reversed yet complimentary fluorescent responses. (B) Plot of RFU normalized by RFU^max^ for 25 nM RAZUL S358C^Acr^ (blue) and 25 nM RAZUL S337C^Atto^ (red) in a common well against varying amounts of AZUL in 10 mM MOPS, pH 6.5, 150 mM NaCl, 5 mM DTT, 10 μM ZnSO_4_, and 0.1% Tween 20. (C) Plot of *K*_d_ values of AZUL binding based on fluorescence measurements of RAZUL S358C^Acr^ (blue) and RAZUL S337C^Atto^ (red) after 0.25, 2, or 4 hours storage at 4 °C for a mixture of 25 nM RAZUL S358C^Acr^ and 25 nM RAZUL S337C^Atto^, with the 0.25 hour timepoint displayed in (B). For (B) and (C), each data point is represented as mean ± SEM. (D) RFU for 100 nM S358C^Acr^ (blue, left), S337C^Atto^ (red, right), and E6AP AZUL following incubation with DMSO or 50 μM RAZUL^322–366^ in 10 mM MOPS, pH 6.5, 150 mM NaCl, 5 mM DTT, 10 μM ZnSO_4_, 0.1% Tween 20, 1% BSA, and 5% DMSO (*n* = 154). (E) Ratiometric RFU value with S358C^Acr^ or S337C^Atto^ in the numerator or denominator as indicated (*n* = 154). In (B) and (C), plots were generated by GraphPad Prism and fit by dose response with variable slope. In (B) through (E), Acr signal is the fluorescence measured with an excitation of 390 nm and emission of 475 nm, whereas Atto signal is measured with an excitation and emission of 580 nm and 632 nm, respectively.

Both RAZUL S337C^Atto^ and RAZUL S358C^Acr^ achieved adequate 
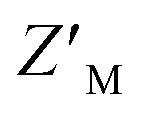
 factors as single fluorescent conformational switch probes, but their differential spectral properties and responses upon AZUL binding led us to explore an assay that measured response with RAZUL S358C^Acr^ and RAZUL S337C^Atto^ present in the same well. We hypothesized that the incorporation of two fluorescent sensors that excite and emit at different wavelengths in the same well (Fig. 6A) could reduce false positives due to screening compound interference (quenching or autofluorescence).

### Dual monitoring of RAZUL S358C^Acr^ and RAZUL S337C^Atto^ achieves excellent robustness, enabling HTP screening

**Fig. 7:**
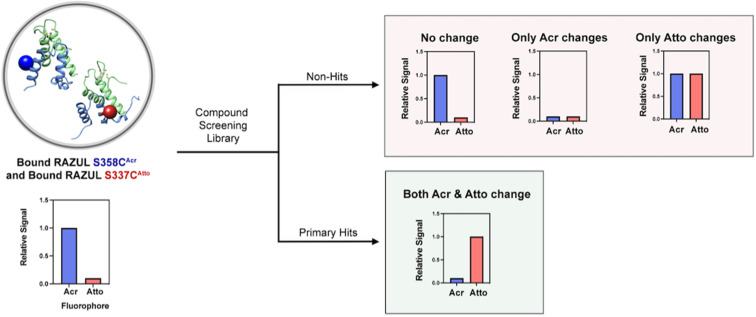
Model of a dually orthogonal assay with RAZUL S358C^Acr^/S337C^Atto^ for elimination of false positives in a HTP screening assay that includes compounds with intrinsic fluorescent properties. Bound RAZUL S358C^Acr^/S337C^Atto^ begins with high fluorescence in the acrylodan (ex. 390 nm; em. 475 nm) channel and low fluorescence in the Atto610 channel (ex. 580 nm; em. 632 nm). After introduction of a compound, a primary hit that inhibits the RAZUL:AZUL interaction will exhibit concomitant responses such that fluorescence of acrylodan decreases and Atto610 increases. Non-hits will elicit no change or only change one of the fluorescence channels.

To determine whether RAZUL S358C^Acr^ and RAZUL S337C^Atto^ can detect the E6AP-induced conformational switch simultaneously, the RFU normalized to RFU^max^ for 25 nM of a mixture of each fluorescently labeled RAZUL domain was measured and plotted with varying concentrations of E6AP AZUL (Fig. 6B and C). In this dually orthogonal assay, RAZUL S358C^Acr^ and RAZUL S337C^Atto^ obtained inflection points of 2.97 ± 0.22 nM and 16.3 ± 1.2 nM at the 0.25 h timepoint, respectively. By ITC, RAZUL S358C^Acr^:AZUL (Fig. 2A) was measured to have a similar affinity to RAZUL S337C^Atto^:AZUL (Fig. 4C), but in a common sample, AZUL bound preferentially to RAZUL S358C^Acr^ over RAZUL S337C^Atto^. The increase in the fluorescence of RAZUL S358C^Acr^ with concomitant decrease for RAZUL S337C^Atto^ following AZUL binding indicates that an inhibitory molecule would decrease S358C^Acr^ fluorescence while increasing S337C^Atto^ fluorescence at their respective excitation/emission wavelengths (Fig. 6A, bottom panel). With a positive control of 50 μM RAZUL^322–366^ and DMSO as a negative control, the 
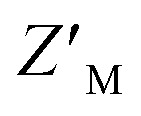
 factor of RAZUL S358C^Acr^ and RAZUL S337C^Atto^ were measured to be 0.74 and 0.54, respectively (Fig. 6D). Comparison of how RAZUL S358C^Acr^ (Fig. 3C) and S337C^Atto^ (Fig. 5E) perform individually *versus* in a common assay (Fig. 6D) indicates the RAZUL S358C^Acr^
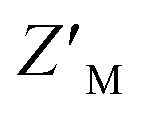
 factor to increase from 0.63 to 0.74 while that of RAZUL S337C^Atto^ slightly decreased from 0.57 to 0.54. Since these fluorophores monitor the RAZUL conformational switch in the same sample, a comparison within each well for RAZUL S358C^Acr^ and RAZUL S337C^Atto^ can be computed to obtain a ratiometric 
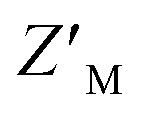
 factor for RAZUL S337C^Atto^/S358C^Acr^ or S358C^Acr^/S337C^Atto^ of 0.73 (Fig. 6E, left panel) or 0.75 (Fig. 6E, right panel), respectively. These values indicate excellent robustness, as required for large scale HTP applications. Thus, this dually orthogonal assay is appropriate for HTP screening, and the combination of two fluorescent sensors will reduce false positives (Fig. 7) to aid in identification of true primary hits.

## Conclusions

Here, we describe the design and development of a dually orthogonal fluorescent-based assay that can be used in a HTP screening campaign for Rpn10:E6AP chemical probes. Rpn10 RAZUL exhibits a disorder-to-order transition when associating with E6AP AZUL, and this conformational switch is captured with acrylodan- and Atto610-labeled Rpn10 RAZUL. Three RAZUL serines were cysteine-substituted to allow for nucleophilic addition to the acrylodan α,β-unsaturated ketone or Atto610 maleimide, with RAZUL S358C and S337C identified as the respective optimal positions. All acrylodan and Atto610 labeled RAZUL probes were sensitive to AZUL binding, but desirable placement for high signal-to-noise ratio was found to be fluorophore-dependent and affinity-independent, indicating local environmental reorientation dictates the extent of spectral change. Previous applications of acrylodan-based screening assays focused on the solvent-exposure of allosteric sites for kinases^50–55^ and phosphatases.^56^ Acrylodan-labeling of intrinsically disordered tau also creatively provided insight into how tau assembles into tubulin dimers, with major spectral hypsochromic shifts indicating that acrylodan was in a more hydrophobic environment.^35^ The greater hypsochromic shift of acrylodan at RAZUL S358C compared to S337C^Acr^ and S361C^Acr^ suggests that when at position 358, acrylodan is directed towards the RAZUL:AZUL interface. Similarly, the significant fluorescence change with Atto610 at RAZUL S337C and not when at S358C or S361C suggests that Atto610 is directed towards the RAZUL:AZUL interface when at position 337. The disparate location for optimal response to AZUL binding could originate from the longer linker region used with the Atto610 fluorophore compared to acrylodan (Fig. S2B and S4B,† respectively).

Competition with unlabeled RAZUL^322–366^ indicated individual 
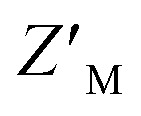
 factors ranging from 0.35–0.63; however, the unique spectral signatures and response of RAZUL S358C^Acr^/S337C^Atto^ binding to AZUL enabled their application in a common sample and a resulting ratiometric 
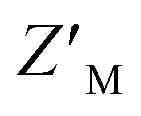
 factor of 0.75. Molecules identified in future screening campaigns with this assay may bind to either RAZUL or AZUL. RAZUL-binding compounds could be rationally designed for direct 26S proteasome recruitment of a target substrate, akin to conjugating a 19S RP Rpn1-binding ligand to a potent BRD4 ligand for targeted protein degradation,^57^ whereas AZUL-binding compounds can be utilized to interrogate E6AP's function at Rpn10, the proteasome, or its other binding partners, such as UBQLN.^58^ Finally, our assay could be modified to identify molecular glues for Rpn10:E6AP, which could be therapeutically beneficial in diseases where mutations lessen their affinity.^28^

Despite the anticipated application of this dually orthogonal Rpn10:E6AP assay, there are inherent limitations, including the slow rate of dissociation (2.11 × 10^−2^ s^−1^)^26^ and <20 nM binding affinity of the native protein–protein interaction, making detection of inhibitors with micromolar affinity challenging. This limitation could be overcome however by making structure-based amino acid substitutions in either RAZUL or AZUL that weaken their binding affinity. Additionally, since this assay depends on the helical formation of Rpn10 RAZUL, screening molecules that non-specifically disrupt helicity would fall into the false positive pool. Thus, as with most screening assays, primary hits will require secondary validation by other biophysical or structural techniques.

To biochemically apply our approach to conformational switches, induced folding (or loss of folding) can be confirmed with the native and modified protein systems prior to fluorescent labeling through integrative biophysical/computational techniques such as CD,^26,59^ NMR,^26,60,61^ small X-ray light scattering (SAXS),^62,63^ single-molecule fluorescence resonance energy transfer (smFRET),^64,65^ and/or molecular modeling.^66,67^ Then, binding affinity retention with the unlabeled/fluorescently labeled proteins and their binding partner can be investigated through the suitable biophysical methods, such as NMR, ITC, SPR, and/or biolayer interferometry (BLI).^68^ If these methods are not accessible, however, acrylodan or Atto610 labeling of a disordered protein-of-interest with high efficiency, as described here, and monitoring fluorescent response under varied conditions can be investigated in 96-well and 384-well plate format. Like Rpn10 RAZUL, there are several therapeutically-relevant proteins that are intrinsically disordered, contain disordered domains, and/or exhibit extensive folding upon binding to a partner.^69–72^ An attractive cancer target that contains disordered regions is tumor suppressor p53,^43,44^ and we expect that environmentally sensitive fluorophores could be applied to discover p53-binding compounds and/or compounds that disrupt the E6-driven interaction of E6AP with p53. As we illustrate here with the RAZUL:AZUL high affinity interaction, the use of environmentally sensitive fluorophores presents screening opportunities for protein systems that exhibit such ubiquitous disorder-to-order (or *vice versa*) transitions.

## Data availability

All data are available in the main text or the ESI.†

## Author contributions

C. S. M., S. G. T., and K. J. W. designed the studies. C. S. M. performed protein expression, purification, labeling, and plate reader assay experiments. C. S. M. and S. G. T. conducted and analyzed CD, fluorimeter, and isothermal titration calorimetry experiments. C. S. M. and K. J. W. wrote the initial draft of the manuscript, and all authors contributed to review and editing of the final manuscript.

## Conflicts of interest

The authors declare no competing interests.

## Supplementary Material

SC-015-D3SC06370D-s001
