# Supplementary material for: High-throughput assay exploiting disorder-to-order conformational switches: application to the proteasomal Rpn10:E6AP complex
Source: Chem Sci. 2024 Feb 6;15(11):4041–53. doi: 10.1039/d3sc06370d (PMC10935766; doi:10.1039/d3sc06370d)
Supplement: SC-015-D3SC06370D-s001 [file SC-015-D3SC06370D-s001.pdf]

Electronic Supplementary Material (ESI) for Chemical Science.
This journal is © The Royal Society of Chemistry 2024

Electronic Supplementary Information for

High-throughput assay exploiting disorder-to-order conformational switches: application to the proteasomal Rpn10:E6AP complex

Christine S. Muli[a], Sergey G. Tarasov[b], Kylie J. Walters[a,*]

[a]Protein Processing Section, Center for Structural Biology, Center for Cancer Research, National Cancer Institute, National Institutes of Health, Frederick, MD, 21702, USA

[b]Biophysics Resource, Center for Structural Biology, Center for Cancer Research, National Cancer Institute, National Institutes of Health, Frederick, MD 21702, USA

[*]Corresponding author email: kylie.walters@nih.gov

Table of Contents

Supplementary Figures & Captions 3
Figure S1 3
Figure S2 5
Figure S3 7
Figure S4. 8
Figure S5. 10
Figure S6 12
Figure S7 14
Methods Details 15
Protein expression and purification 15
Acrylodan-labeling of cysteine-substituted Rpn10 RAZUL 16
Atto610- and DY647P1-labeling of cysteine-substituted Rpn10 RAZUL 16
LC-MS collection and analysis of labeled mutants 17
Circular dichroism (CD) spectroscopy 17
Isothermal titration calorimetry (ITC) 18
Fluorescence spectroscopy with FluoroMax-4C 18
Fluorescence assays in 96-well and 384-well plates 19
Measurements/calculations for fluorescently labeled Rpn10 RAZUL assays 19
Assay quality metrics 20
References 22

Supplementary Figures & Captions

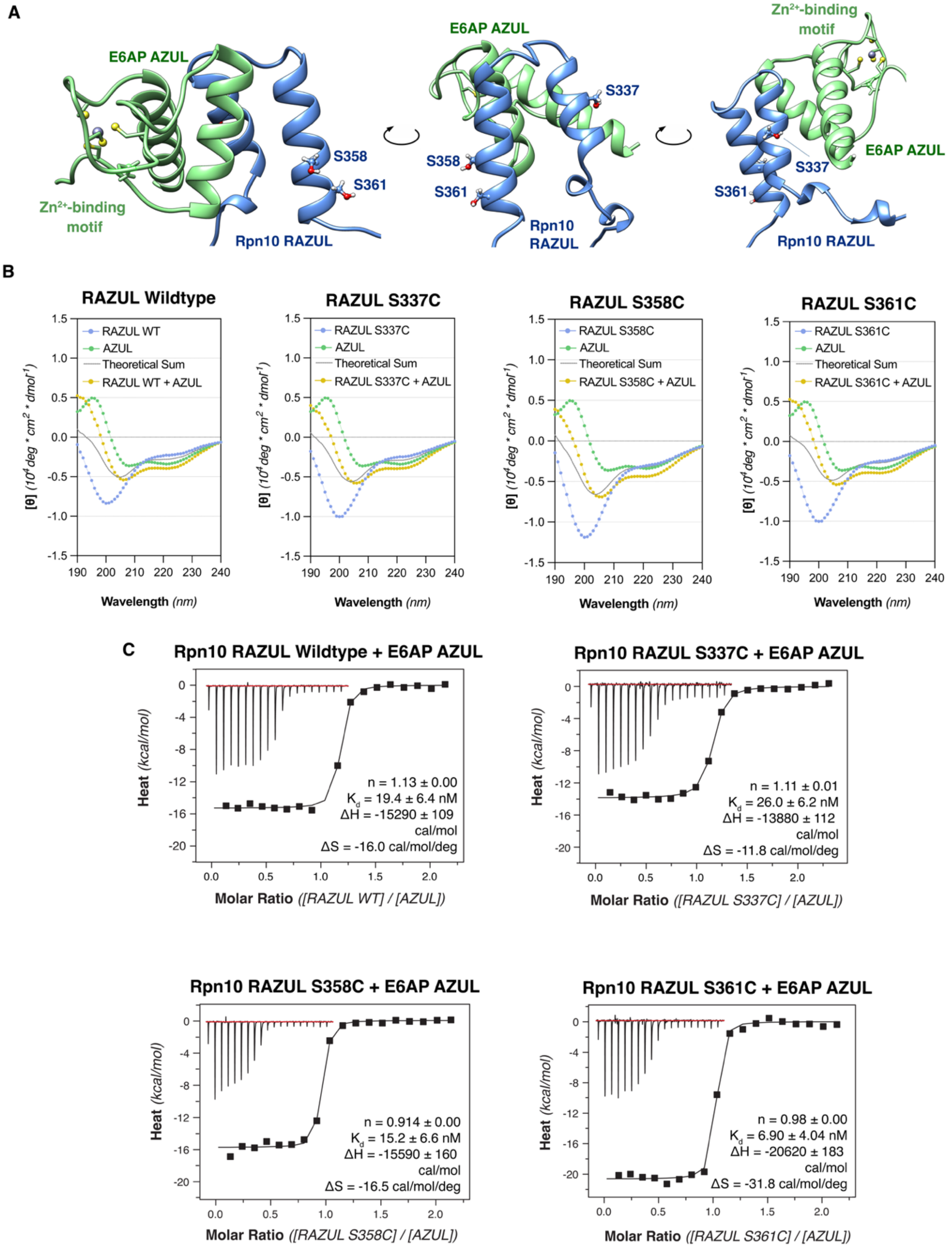

Figure S1

Figure S1. Cysteine-substitution at RAZUL codon 337, 358, or 361 does not disrupt AZUL binding. (A) Ribbon diagrams of the Rpn10 RAZUL (blue):E6AP AZUL (green) complex highlighting three native serines (S337, S358, and S361, ball-and-stick heavy sidechain atoms) proximal to the intermolecular contact surface. Sidechain heavy atoms are shown for Zn^{2+}-coordinating cysteines. Oxygen, hydrogen, and sulfur are colored red, white, and yellow, respectively. PDB: 6U19. **(B)** CD spectra recorded on 25 μM of purified unmodified or serine-to-cysteine mutated RAZUL (blue), E6AP AZUL (green), or the equimolar mixture (yellow) as indicated with inclusion of the theoretical sum (gray). Experiments were performed in 10 mM Na_2HPO_4, pH 6.5, 10 mM NaF, 1 mM TCEP, and 10 μM $ZnSO_4$. **(C)** Binding isotherm with raw ITC data (top insert) for injections of 170 mM E6AP AZUL into 18 mM wildtype or mutated Rpn10 RAZUL in 10 mM MOPS, pH 6.5, 50 mM NaCl, 2 mM TCEP, and 10 μM $ZnSO_4$. Fitted thermodynamic values are included (lower right): n, stoichiometry; K_d, binding affinity; DH, change in enthalpy; DS, change in entropy.

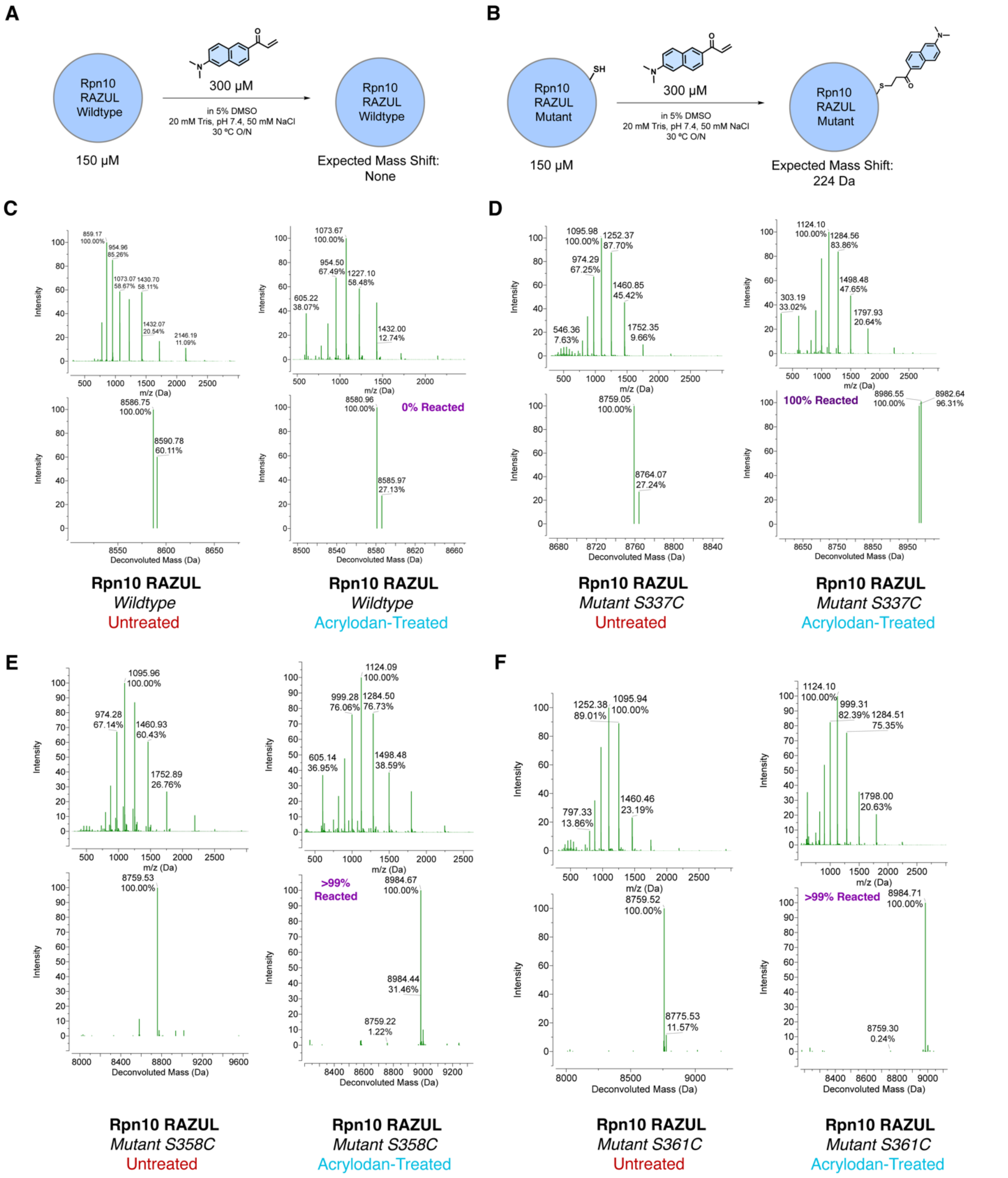

Figure S2. Acrylodan labeling of cysteine-substituted Rpn10 RAZUL was achieved to >99% efficiency. (A – B) Reaction schemes of acrylodan (fluorophore in blue) with (A) wildtype or (B) cysteine-substituted Rpn10 RAZUL. 150 µM of Rpn10 RAZUL is reacted with 300 µM acrylodan in 20 mM Tris, pH 7.4, 50 mM NaCl (supplemented with 5% DMSO) for 16 – 20 hours overnight (O/N) at 30 ºC. Wildtype RAZUL, which has no native cysteines and an expected mass of 8,586

Da, should have no reaction. Cysteine-substituted RAZUL mutants, which have an expected mass of 8,759, would acquire a 224 Da mass shift when acrylodan-labeled. **(C – F)** LC-MS spectra (top) with deconvolution (bottom) for Rpn10 RAZUL (C) wildtype, (D) S337C, (E) S358C, or (F) S361C without (left) or with (right) acrylodan treatment. Protein deconvolution was done by MestReNova.

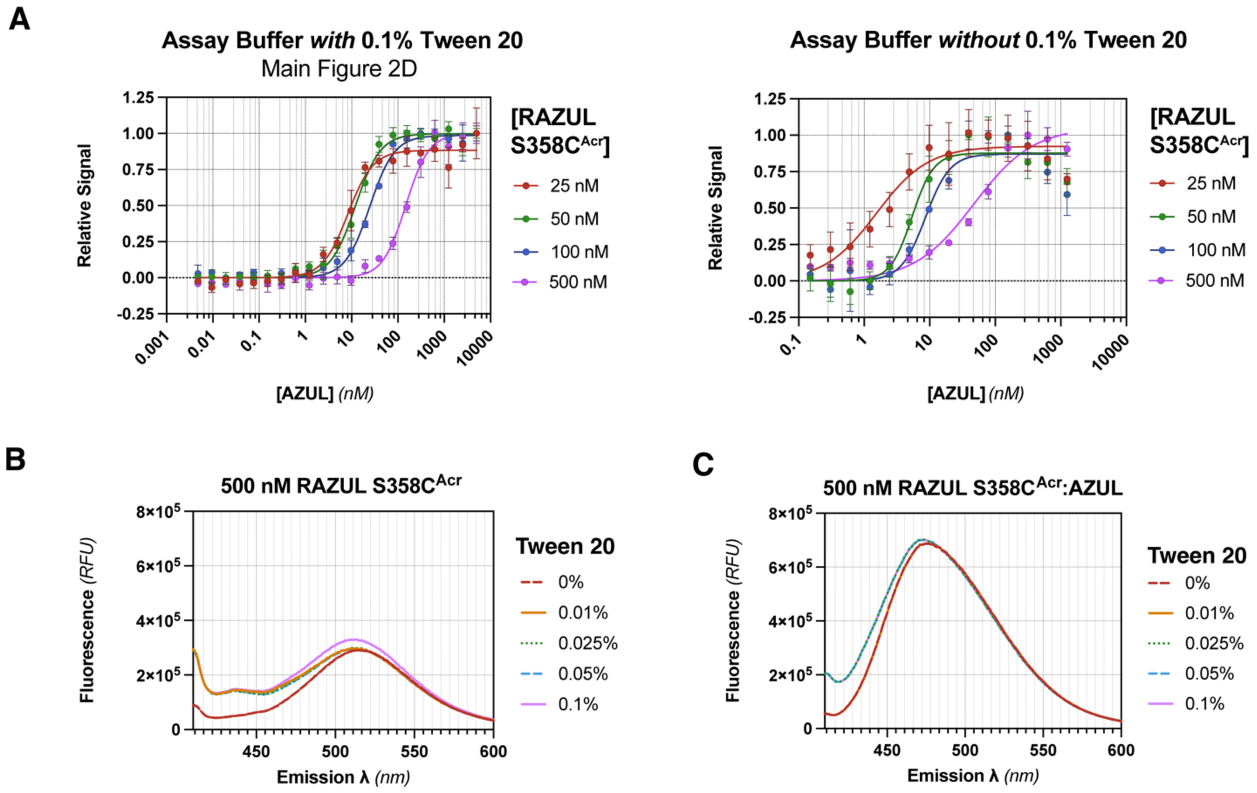

Figure S3. Detergent reduces aggregation in a plate reader assay without significant effect on fluorescent properties. **(A)** RAZUL S358C^{Acr} binding curves in 384-well plates with E6AP AZUL where buffer includes (left, same as Figure 2D) or lacks (right) 0.1% Tween 20. A constant concentration of RAZUL S358C^{Acr} [25 nM (red), 50 nM (green), 100 nM (blue), or 500 nM (purple)] was tested with increasing concentrations of AZUL in 10 mM MOPS, 50 mM NaCl, 5 mM DTT, and 10 μM $ZnSO_4$. Results were plotted by GraphPad Prism and fit to a Hill curve for specific binding. **(B – C)** Fluorescence spectroscopy of 500 nM RAZUL S358C^{Acr} in 10 mM MOPS, pH 6.5, 50 mM NaCl, 5 mM DTT, 10 μM $ZnSO_4$ with 0% (red dashed), 0.01% (orange solid), 0.025% (green dotted), 0.05% (blue dashed), and 0.1% (purple line) Tween 20 (B) without and (C) with 500 nM AZUL. All samples were excited on a FluoroMax-4C spectrofluorometer at 390 nm with emission scanning range from 410 – 600 nm.

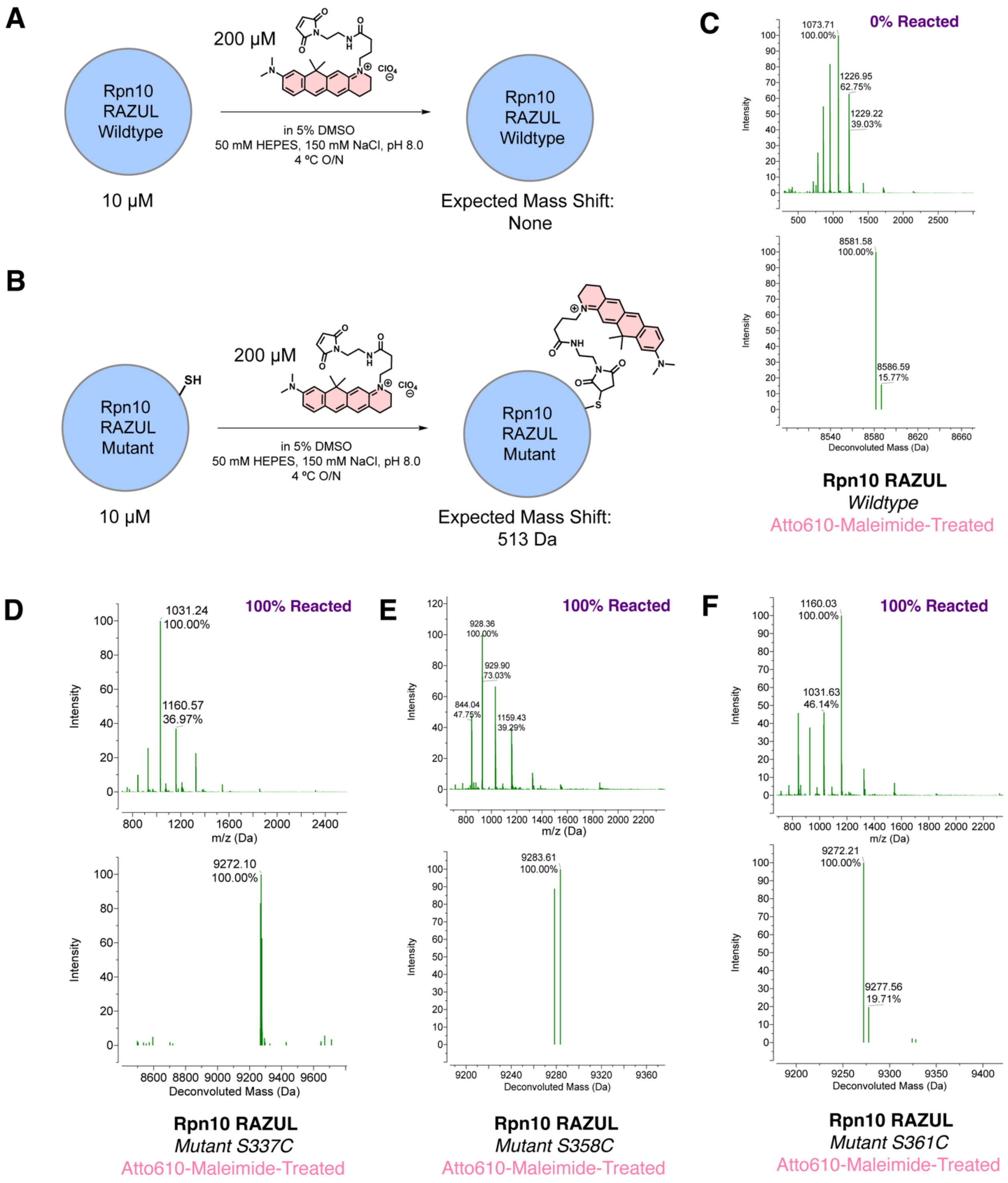

Figure S4. Atto610 labeling of cysteine-substituted Rpn10 RAZUL achieved >99% efficiency. (A – B) Reaction schemes of Atto610-maleimide with Rpn10 RAZUL (A) wildtype or (B) serine-to-cysteine mutant. 10 μM of Rpn10 RAZUL is reacted with 200 μM Atto610-maleimide

in 50 mM HEPES, pH 8.0, 150 mM NaCl (supplemented with 5% DMSO) for 16 – 20 hours overnight (O/N) at 4 ºC. Wildtype RAZUL, which has no native cysteines and an expected mass of 8,586 Da, should have no reaction. Mutants, which have an expected mass of 8,759, would acquire a 513 Da mass shift when Atto610-labeled. **(C – F)** LC-MS spectra (top) with deconvolution (bottom) for Rpn10 RAZUL (C) wildtype, (D) S337C, (E) S358C, or (F) S361C after Atto610-maleimide treatment. Protein deconvolution was done by MestReNova.

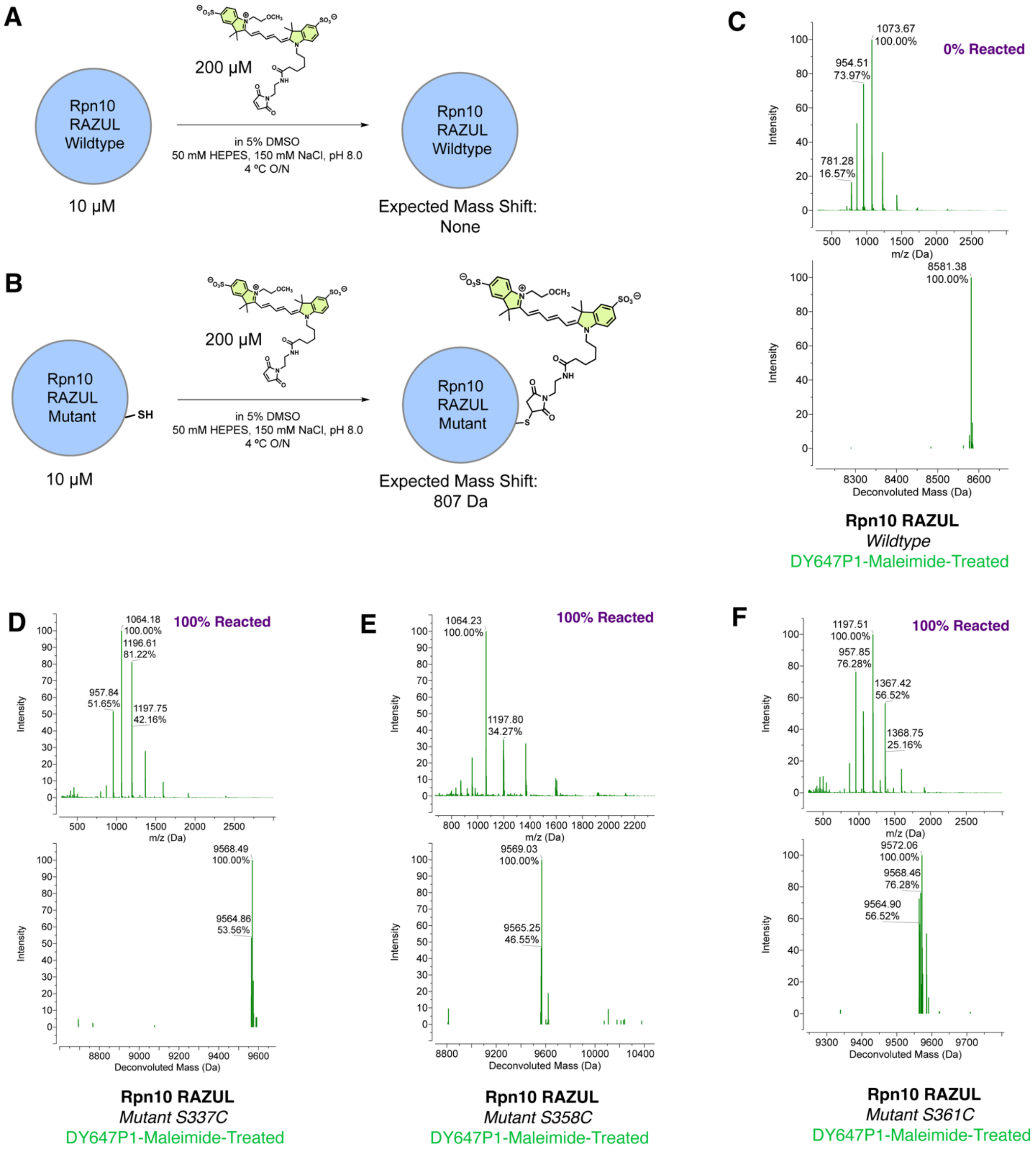

Figure S5. DY647P1 labeling of cysteine-substituted Rpn10 RAZUL was achieved at >99% efficiency. (A – B) Reaction schemes of DY647P1-maleimide with Rpn10 RAZUL (A) wildtype or (B) serine-to-cysteine mutant. 10 µM of Rpn10 RAZUL is reacted with 200 µM DY647P1-maleimide in 50 mM HEPES, pH 8.0, 150 mM NaCl (supplemented with 5% DMSO) for 16 – 20 hours overnight (O/N) at 4 ºC. Wildtype RAZUL, which has no native cysteines and an expected

mass of 8,586 Da, should have no reaction. Mutants, which have an expected mass of 8,759, would acquire an 807 Da mass shift when DY647P1-labeled. **(C – F)** LC-MS spectra (top) with deconvolution (bottom) for Rpn10 RAZUL (C) wildtype, (D) S337C, (E) S358C, or (F) S361C after DY647P1-maleimide treatment. Protein deconvolution was done by MestReNova.

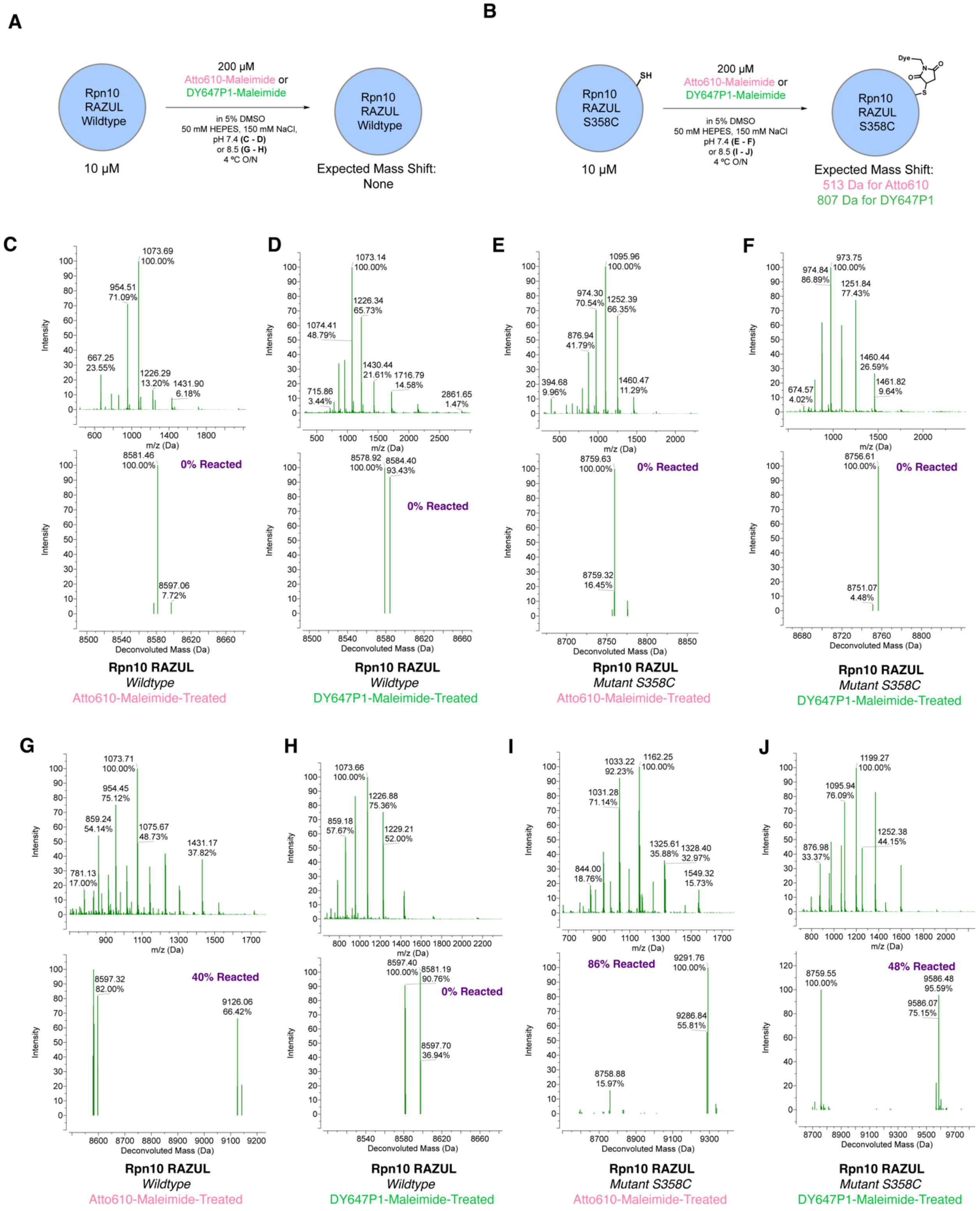

Figure S6. Dye-maleimides ineffectively or non-specifically labeled cysteine-substituted Rpn10 RAZUL S358C at pH 7.4 or 8.5, respectively. **(A – B)** Reaction schemes of dye-

maleimide with Rpn10 RAZUL (A) wildtype and (B) S358C with buffer at pH 7.4 or 8.5. 10 µM of Rpn10 RAZUL is reacted with 200 µM dye-maleimide in 50 mM HEPES, pH 7.4 or 8.5, 150 mM NaCl (supplemented with 5% DMSO) for 16 – 20 hours overnight (O/N) at 4 ºC. Unreacted wildtype and S358C RAZUL are expected to have a deconvoluted mass of 8,581 and 8,759 Da, respectively. **(C – F)** Reaction outcomes at pH 7.4. LC-MS spectra (top) with deconvolution (bottom) for Rpn10 RAZUL (C) wildtype with Atto610-maleimide, (D) wildtype with DY647P1-maleimide, (E) S358C with Atto610-maleimide, or (F) S358C with DY647P1-maleimide treatment. **(G – J)** Reaction outcomes at pH 8.5. LC-MS spectra (top) with deconvolution (bottom) for Rpn10 RAZUL (G) wildtype with Atto610-maleimide, (H) wildtype with DY647P1-maleimide, (I) S358C with Atto610-maleimide, or (J) S358C with DY647P1-maleimide treatment. Protein deconvolution was done by MestReNova.

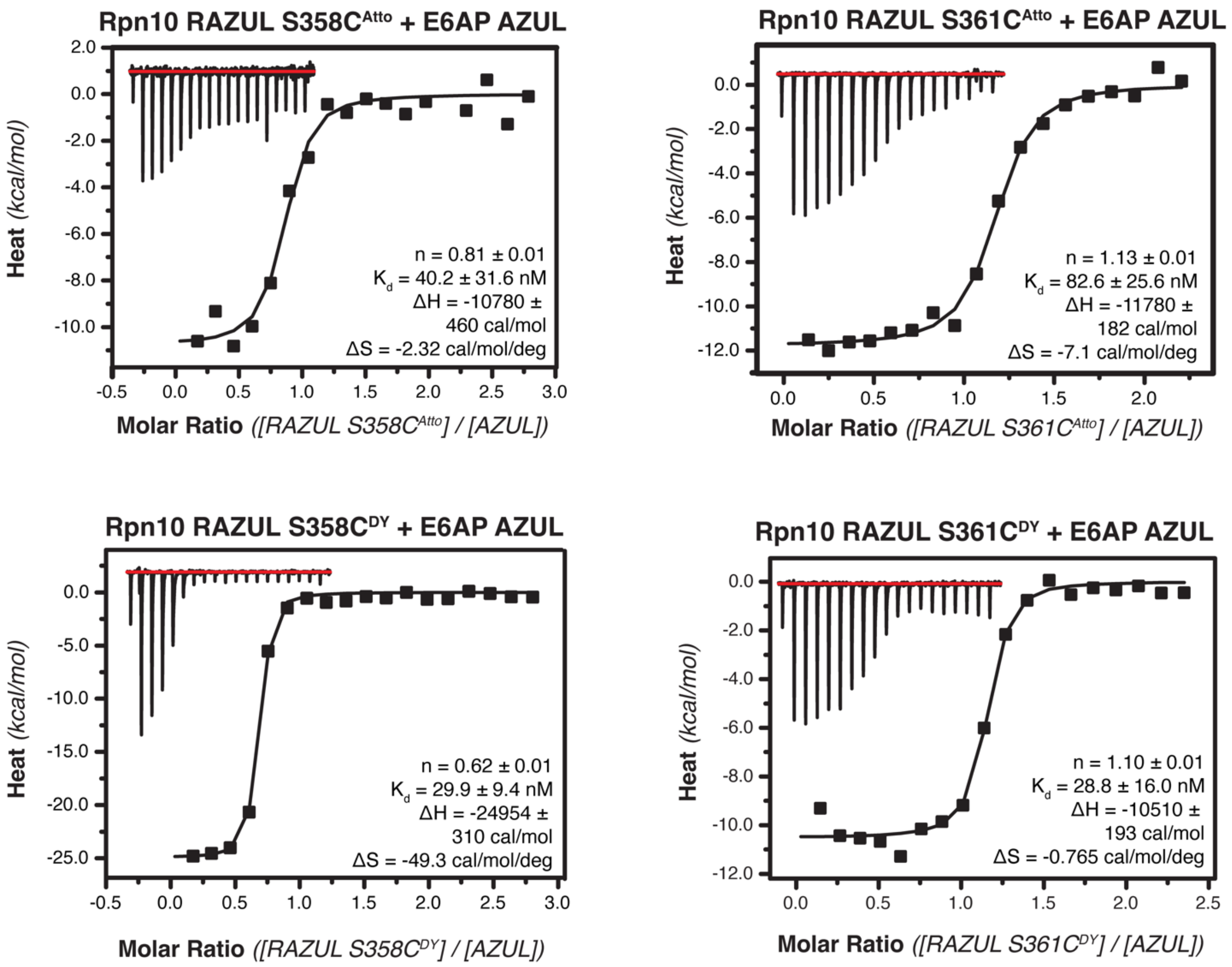

Figure S7. Atto610 and DY647P1 labeled Rpn10 RAZUL mutants retain binding to E6AP AZUL by ITC. Binding isotherm with raw ITC data (top left insert) for injections of AZUL into Atto610-labeled (top) or DY647P1-labeled (bottom) Rpn10 RAZUL at 40 µM AZUL into 3 µM RAZUL S358C^{Atto} (top left panel), 95 µM AZUL into 9 µM RAZUL S361C^{Atto} (top right panel), 157 µM AZUL into 12 µM RAZUL S358C^{DY} (bottom left panel), and 90 µM AZUL into 8 µM RAZUL S361C^{DY} (bottom right panel). Fitted thermodynamic values are included on each panel at the bottom right: n, stoichiometry; K_d, binding affinity; ΔH, change in enthalpy; ΔS, change in entropy. Measurements were made in 10 mM MOPS, pH 6.5, 50 mM NaCl, 2 mM TCEP, and 10 µM $ZnSO_4$. This figure relates to Figure 4C.

Methods Details

Protein expression and purification

The following constructs were purchased from GenScript with codon optimization for expression in *E. coli*: Rpn10 (305-377 S337C), Rpn10 (305-377 S358C), and Rpn10 (305-377 S361C). The cDNAs were inserted into a pGEX-6P-1 vector between EcoRI and XhoI sites in frame with an N-terminal glutathione S-transferase (GST) and a PreScission Protease cleavage site. Wildtype Rpn10 (305-377) and E6AP (24-87) were subcloned as previously described.[1]

Rpn10 RAZUL plasmids were transformed into *E. coli* strain BL21 (DE3) (Thermo Fisher Scientific C600003) supplemented with ampicillin, whereas E6AP AZUL was transformed into *E. coli* strain BL21 (DE3) with kanamycin selection. Transformed cells were grown at 37 ºC in LB broth (supplemented with ampicillin for Rpn10 RAZUL or kanamycin for E6AP AZUL) to an OD_{600} of 0.5 – 0.6 and induced with 0.5 mM IPTG at 18 ºC overnight for 16 – 20 hours. Bacteria were pelleted by centrifugation at 4000 rpm at 4 ºC for 40 minutes using a Beckman Coulter J6-M1 centrifuge with a JS-4.2 rotor. Pellets were resuspended in Buffer A (10 mM MOPS, pH 6.5, 50 mM NaCl, 5 mM DTT, 10 µM $ZnSO_4$) supplemented with Roche EDTA-free protease inhibitor cocktail (Roche Diagnostics 11836170001). Resuspended bacteria were lysed with sonication at 4 ºC and centrifuged at 20,000 rpm and 4 ºC for 30 minutes using a Beckman Avanti J-25 I centrifuge with a JA-25.50 rotor.

For GST-tagged Rpn10 RAZUL wildtype and mutants, supernatants were incubated with pre-washed glutathione sepharose beads (Cytiva 17-0756-05) for 1 hour at room temperature. Beads were washed with Buffer A three times. Rpn10 RAZUL was cleaved from GST on-bead overnight at 4 ºC with PreScission Protease (Cytiva 270884301) in Buffer B (50 mM Tris-HCl, pH 7.0, 150 mM NaCl, 1 mM EDTA, 1 mM DTT). For His-tagged E6AP AZUL, the supernatant was incubated with pre-washed Ni-NTA beads (Qiagen 30210) for 1 hour at room temperature and washed three times with Buffer A. E6AP AZUL was released from the His-tag on-bead overnight at 4 ºC with thrombin protease (EMD Millipore 605195) in Buffer C [1X Phosphate Buffered Saline (PBS) or 10 mM Na_2HPO_4, 1.8 mM KH_2PO_4, 2.7 mM KCl, 137 mM NaCl, pH 7.4].

Eluted cleaved proteins were purified by size exclusion chromatography on an FPLC ÄKTA pure (GE Healthcare) using a HiLoad 16/600 Superdex 75 in Buffer A. Purity of protein was assessed by SDS-PAGE, and samples were dialyzed in Buffer A at 4 ºC prior to use. If buffer exchange was

required, samples were buffer exchanged with columns (Thermo Fisher Cat. No. 89882) or dialyzed prior to experimental application.

Acrylodan-labeling of cysteine-substituted Rpn10 RAZUL

Cysteine-substituted Rpn10 RAZUL mutants were buffer exchanged into Buffer D (20 mM Tris, pH 7.4, 50 mM NaCl) and prepared at 157.8 µM. Acrylodan (Thermo Fisher Cat. No. A433) was prepared in DMSO at 6000 µM. At a 200 µL scale, 190 mL of 157.8 µM protein and 10 µL of 6000 µM dye were combined. Overall, this reaction was performed at a 1:2 protein:dye ratio with 150 µM protein and 300 µM dye. Samples were agitated at 30 ºC overnight and protected from light. Unbound excess dye was removed by buffer exchange into Buffer A four to five times. Labeling of RAZUL mutants with acrylodan was confirmed by LC-MS analysis, **Figure S2.**

Deconvolution of multiply charged states of RAZUL was processed by MestReNova. Percent labeling was approximated with the **Equation 1** with the assumption that unlabeled and labeled RAZUL ionize similarly.

$$\%\ Labeled = \frac{RAZUL_{Labeled}}{RAZUL_{Unlabeled} + RAZUL_{Labeled}}\ x\ 100\% \quad (1)$$

As a control, Rpn10 RAZUL wildtype was buffer exchanged into Buffer D and treated with acrylodan under the same conditions. Excess dye was removed, and sample was analyzed by LC-MS to ensure no reactivity, **Figure S2C.**

Atto610- and DY647P1-labeling of cysteine-substituted Rpn10 RAZUL

Cysteine-substituted Rpn10 RAZUL mutants were buffer exchanged into Buffer E (50 mM HEPES, pH 8.0, 150 mM NaCl) and prepared at 10.52 µM. Atto610 maleimide (Atto-tec Cat. No. AD 610-45) and DY647P1 maleimide (Dyomics Cat. No. 647P1-03) were prepared in DMSO at 4000 µM. At a 1 mL scale, 950 mL of 10.52 µM protein and 50 µL of 4000 µM dye were combined. Overall, this reaction was performed at a 1:20 protein:dye ratio with 10 µM protein and 200 µM dye. Samples were rotated at 4 ºC overnight (16 – 20 hours) and protected from light. Unbound excess dye was removed by buffer exchange into Buffer A four to five times. Labeling of RAZUL mutants with Atto610 or DY647P1 was confirmed by LC-MS analysis, **Figure S4 – S5**, respectively**.**

Percent labeling was approximated with the Equation 1 with the assumption that unlabeled and labeled RAZUL ionize similarly. As a control, Rpn10 RAZUL wildtype was buffer exchanged into Buffer E and treated with Atto610/DY647P1 under the same conditions. Excess dye was removed, and sample was analyzed by LC-MS to ensure no reactivity, **Figure S4C** and **S5C.**

LC-MS collection and analysis of labeled mutants

10 μM of labeled protein (or unlabeled protein for control samples) was prepared in 50/50 water/acetonitrile for LC-MS collection and analysis. Mass spectrometry data were acquired on an Agilent 6130 Quadrupole LC-MS System, (Agilent Technologies, Inc.) equipped with electro-spray source, operated in positive-ion mode. Separation was performed on a 300SB-C3 Poroshell column (2.1 mm x 75 mm; particle size 5 μm). Approximately 25 pmol of protein was injected and were eluted at a flow rate of 1 mL/min with a 5 - 100% increase of mobile phase B over 5 minutes and holding with mobile phase B for 1 minute. Mobile phase A contained 5% acetic acid in water, and mobile phase B was acetonitrile.

Mass spectra were analyzed and deconvoluted with MestReNova software. Full protein peaks were analyzed and deconvoluted with tolerance set to 50 –150 ppm, abundance threshold at 1%, charged state range from 4 – 11, and mass-to-charge range from 600 – 2300 Da. For each labeled sample, deconvoluted mass range varied: 8000 – 9500 Da for acrylodan-labeling, 8500 – 10200 Da for Atto610-labeling, and 8500 – 10400 Da for DY647P1-labeling. Spectra were also checked for doubly-labeled proteins, but double addition of dye was not observed.

Circular dichroism (CD) spectroscopy

Far-UV range circular dichroism (CD) spectra (240 – 190 nm) of 25 μM Rpn10 RAZUL (wildtype or mutant), 25 μM E6AP AZUL, and the mixture of 25 μM Rpn10 RAZUL (wildtype or mutant) and 25 μM E6AP AZUL were recorded on a Jasco J-1500 CD spectrometer using a quartz cuvette with a 1.0 mm path length (Hellma Analytics, Cat. No. 110-1-40) and controlled temperature of 25 ºC. Samples were buffer exchanged into Buffer F (10 mM Na_2HPO_4, 10 mM NaF, 1 mM TCEP, and 10 μM $ZnSO_4$, pH 6.5). All spectra were collected continuously at a scan speed of 50 nm/min and averaged over accumulation of three spectra. The molar ellipticity (θ) was calculated from the raw millidegrees (m^o) at wavelength lambda using **Equation 2**:

$$\theta = \frac{m^o}{10 * C * L * \#AA} \tag{2}$$

where C is the concentration of the sample in mol L^{-1}, L is the path length of the cell in cm, and number of amino acids (# AA) for RAZUL is 72 and AZUL is 63.

Isothermal titration calorimetry (ITC)

ITC was performed at 25 °C on a MicroCal iTC200 system (Malvern Panalytical). Rpn10 RAZUL (wildtype protein and unlabeled/labeled mutants) were extensively co-dialyzed with E6AP AZUL in Buffer G (10 mM MOPS, pH 6.5, 50 mM NaCl, 2 mM TCEP, and 10 µM $ZnSO_4$. Eighteen 2.1 µL aliquots of E6AP AZUL were injected at 750 rpm into a calorimeter cell (volume 200.7 µL) that contained 10-fold less concentrated Rpn10 RAZUL. For example, if the titrant E6AP AZUL was 80 µM, Rpn10 RAZUL in the cell was 10-fold less at 8 µM. Reference experiments were performed by replacing protein sample in the cell with Buffer G, and this reference data was subtracted from the experimental data during analysis. The integrated interaction heat values were normalized as a function of the molar ratio of Rpn10 RAZUL to E6AP AZUL, and the data were fit with MicroCal Origin 7.0 software. Binding was assumed to be at one site to yield the binding affinity K_a ($1/K_d$), stoichiometry, and other thermodynamic parameters.

Fluorescence spectroscopy with FluoroMax-4C

Fluorescence emission spectrum of labeled Rpn10 RAZUL constructs with and without E6AP AZUL were recorded on a FluoroMax-4C (HORIBA Scientific) spectrofluorometer using a quartz cell cuvette with a 2 x 10 mm dual path length (Starna Cells, Inc., Cat. No 53-Q-2) at 25 ºC. For acrylodan-labeled Rpn10 RAZUL, samples were excited at 390 nm (slit: 5 nm) and emission scanned from 410 – 600 nm (slit: 5 nm). For Atto610-labeled Rpn10 RAZUL, samples were excited at 590 nm (slit: 4 nm) and emission scanned from 600 – 750 nm (slit: 4 nm). For DY647P1-labeled Rpn10 RAZUL, samples were excited at 630 nm (slit: 3 nm) and emission scanned from 640 – 800 nm (slit: 3 nm). Integration time for all samples was 0.5 seconds.

For **Figures 2B** and **4A,** fluorescence was recorded in Buffer A. For **Figure S3B** and **S3C**, fluorescence was recorded in Buffer A supplemented with 0%, 0.01%, 0.025%, 0.05%, or 0.1% Tween 20.

Fluorescence assays in 96-well and 384-well plates

Fluorescence assays were conducted using a CLARIOstar microplate reader with 96-well (Corning, Black Flat Bottom, Non-Binding Surface, Polystyrene; Cat. No. 3993) or 384-well (Corning, Black Flat Bottom, Non-Binding Surface, Polystyrene; Cat. No. 3575) plates. Final conditions were performed in Buffer A, Buffer I (Buffer A supplemented with 0.1% Tween 20), Buffer J (10 mM MOPS, pH 6.5, 150 mM NaCl, 5 mM DTT, 10 µM $ZnSO_4$, 0.1% Tween 20), or Buffer K (10 mM MOPS, pH 6.5, 300 mM NaCl, 5 mM DTT, 10 µM $ZnSO_4$, 0.1% Tween 20).

For experiments with E6AP AZUL, E6AP AZUL (or negative control, BSA) was serially diluted within the assay plate. Next, a stock of dye labeled Rpn10 RAZUL was added to all wells. The plate was spun down, and measurements were taken immediately. If experiment was time-dependent, samples were protected with foil and stored at 4 ºC prior to measurement. Measurements by CLARIOstar were taken at room temperature.

For competition experiments with unmodified or phosphorylated Rpn10$^{322\text{-}366}$, the peptides were serially diluted within the assay plate, followed by the addition of a stock of E6AP AZUL to all wells. The plate was spun down and stored at 4 ºC for 10 – 15 minutes. After equilibration, a stock of dye-labeled Rpn10 RAZUL was added to all wells. The plate was spun down, and measurements were taken immediately. If experiment was time-dependent, samples were protected with foil and stored at 4 ºC prior to measurement. Measurements by CLARIOstar were taken at room temperature. The final DMSO percentage throughout the assay was maintained at 5% (%v/v).

For Z' factor experiments only, Buffer L (10 mM MOPS, pH 6.5, 150 mM NaCl, 5 mM DTT, 10 µM $ZnSO_4$, 1% BSA, 0.1% Tween 20) was used, with BSA acting as a carrier protein to reduce non-specific interactions. Final DMSO percentage within the assay was at 5% (%v/v). Within the CLARIOstar plate reader, samples were shaken at 300 rpm (double orbital) for 30 seconds prior to measurement.

Measurements/calculations for fluorescently labeled Rpn10 RAZUL assays

Fluorescence intensity measurements to monitor fluorescently labeled RAZUL were performed with an excitation of 390 (± 15) nm and emission of 475 (± 20) or 500 (± 20) nm for acrylodan-labeled RAZUL (RAZUL^{Acr}) , excitation of 580 (± 15) nm and emission of 632 (± 20) nm for Atto610-labeled RAZUL (RAZUL^{Atto}), and an excitation of 617 (± 15) nm and emission of 667 (±

20) nm for DY647P1-labeled RAZUL ($RAZUL^{DY}$). Gain was set by adjusting towards the max signal of the experiment, which was fluorophore-dependent: bound $RAZUL^{Acr}$ or unbound $RAZUL^{Atto}$/$RAZUL^{DY}$.

Relative fluorescent units (RFUs) were calculated by **Equation 3^{Acr}**:

$$x \text{ (in RFU)} = Signal_{measured} - Signal_{background} \quad (3^{Acr})$$

where $Signal_{background}$ is the average of a triplicate measurement of $RAZUL^{Acr}$ in buffer only. For normalization to compare the free and bound state of various assay conditions such that the max relative signal is 1.0, the following **Equation 4** was applied to the RFU calculated in Equation 3.

$$\text{Relative Signal} = x / x_{max} \quad (4)$$

where x is the normalized signal from **Equation 3^{Acr}** of a particular condition and x_{max} is the maximum signal within the titration or experiment. x_{max} is fully bound acrylodan-labeled RAZUL. Data was then plotted on GraphPad Prism 9. For fitting, curves were analyzed with non-linear regression parameters, either the Specific binding with Hill slope or Dose-Response with variable slope (four parameters) with asymmetric confidence intervals.

RFU of $RAZUL^{Atto}$ or $RAZUL^{DY}$ was normalized using **Equation $3^{Atto/DY}$**:

$$x \text{ (in RFU)} = Signal_{bound} - Signal_{measured} \quad (3^{Atto/DY})$$

where $Signal_{bound}$ is the average of a triplicate measurement of $RAZUL^{Atto}$/$RAZUL^{DY}$ with a saturating AZUL concentration. For normalization to compare the free and bound state of various assay conditions, such that the max relative signal is 1.0, **Equation 4 (above)** was applied. In these conditions, x_{max} is unbound $RAZUL^{Atto}$/$RAZUL^{DY}$.

Assay quality metrics

The Z' factor was calculated by **Equation 5** as described previously[2]:

$$Z' = 1 - \frac{3\,(\sigma_p + \sigma_n)}{|\,\mu_p - \mu_n\,|} \quad (5)$$

where μ_p and μ_n are the average signal of the positive (RAZUL[322-366]) and negative (DMSO, vehicle) control, respectively, and σ_p *and* σ_n are the standard deviations of the positive and negative control samples, respectively.

The robust Z' factor (Z'_M) was calculated by **Equation 6** as described previously[3, 4]:

$$Z'_M = 1 - \frac{3\,(MAD_p + MAD_n)}{|\,M_p - M_n\,|} \quad (6)$$

where M_p and M_n are the median signal of the positive (RAZUL[322-366]) and negative (DMSO, vehicle) control, respectively, and MAD_p *and* MAD_n are the median absolute deviations (MAD) of the positive and negative control samples, respectively.

The MAD for each control group was calculated by **Equation 7**:

$$MAD = median\ (|X_i - X|) \quad (7)$$

where X_i is the raw fluorescent value of a well and X is the overall median of the control group

References

(1) Buel, G. R.; Chen, X.; Chari, R.; O'Neill, M. J.; Ebelle, D. L.; Jenkins, C.; Sridharan, V.; Tarasov, S. G.; Tarasova, N. I.; Andresson, T.; Walters, K. J. Structure of E3 ligase E6AP with a proteasome-binding site provided by substrate receptor hRpn10. *Nat Commun* **2020**, *11* (1), 1291. DOI: https://doi.org/10.1038/s41467-020-15073-7.

(2) Zhang, J. H.; Chung, T. D.; Oldenburg, K. R. A Simple Statistical Parameter for Use in Evaluation and Validation of High Throughput Screening Assays. *J Biomol Screen* **1999**, *4* (2), 67-73. DOI: https://doi.org/10.1177/108705719900400206.

(3) Atmaramani, R.; Pancrazio, J. J.; Black, B. J. Adaptation of robust Z' factor for assay quality assessment in microelectrode array based screening using adult dorsal root ganglion neurons. *Journal of Neuroscience Methods* **2020**, *339*, 108699. DOI: https://doi.org/10.1016/j.jneumeth.2020.108699.

(4) Birmingham, A.; Selfors, L. M.; Forster, T.; Wrobel, D.; Kennedy, C. J.; Shanks, E.; Santoyo-Lopez, J.; Dunican, D. J.; Long, A.; Kelleher, D.; et al. Statistical methods for analysis of high-throughput RNA interference screens. *Nat Methods* **2009**, *6* (8), 569-575. DOI: https://doi.org/10.1038/nmeth.1351.
